# RAI2 acts as a tumor suppressor with functional significance in gastric cancer

**DOI:** 10.18632/aging.205135

**Published:** 2023-10-25

**Authors:** Xiaoli Lou, Wei Deng, Lixiong Shuai, Yijing Chen, Mengmeng Xu, Jingze Xu, Yongsheng Zhang, Yongyou Wu, Zhifei Cao

**Affiliations:** 1 Department of Pathology, The Second Affiliated Hospital of Soochow University, Suzhou, P.R. China; 2 Department of Pathology, Kunshan Hospital of Traditional Chinese Medicine, Kunshan, P.R. China; 3 Department of General Surgery, The Second Affiliated Hospital of Soochow University, Suzhou, P.R. China

**Keywords:** RAI2, gastric cancer, PD-L1, immune infiltration, metastasis

## Abstract

Metastasis of gastric cancer (GC) is one of the major causes of death among GC patients. GC metastasis involves numerous biological processes, yet the specific molecular biological mechanisms have not been elucidated. Here, we report a novel tumor suppressor, retinoic acid-induced 2 (RAI2), which is located in the Xp22 region of the chromosome and plays a role in inhibiting GC growth and invasion. In this study, integrated analysis of The Cancer Genome Atlas (TCGA), Gene Expression Omnibus (GEO) datasets and immunohistochemistry staining data suggested that RAI2 expression in GC samples was low. Moreover, the immune infiltration analysis indicated that low expression of RAI2 in GC was associated with a higher intensity of tumor-infiltrating lymphocytes (TILs) and an abundance of Programmed death ligand 1 (PD-L1) expression. Gene set enrichment analysis (GSEA) analysis further revealed that RAI2 regulated some pathways including the GAP junction, focal adhesion and ECM receptor interaction pathway, immune regulation, PI3K-Akt signaling, MAPK signaling, cell cycle, and DNA replication. Furthermore, the knockdown of RAI2 promoted GC cell proliferation, migration, and invasion *in vitro*. Taken together, these results suggest that the tumor suppressor RAI2 could be a potential target for the development of anti-cancer strategies in GC.

## INTRODUCTION

Gastric cancer is a prevalent tumour of the digestive system that threaten human health and has a high incidence rate, especially in Asia [1, 2]. In China, the incidence rate of GC scores second among carcinomas and has the third most powerful mortality rate among all cancer types [3, 4]. The early diagnosis rate of GC in China is still low, and more than 90% of hospitalized cases are progressive GC. Even if radical surgery is performed, the 5-year survival rate still hovers at 30–40%. Metastasis is the main factor affecting the prognosis of GC patients and is the primary cause of late death of GC patients [4–6]. Thus, it is of essential clinical importance to explore the mechanisms of biology underlying GC metastasis and to identify potential molecular and biological targets involved in GC progression in order to prevent and manage GC occurrence and development.

**Figure 1:**
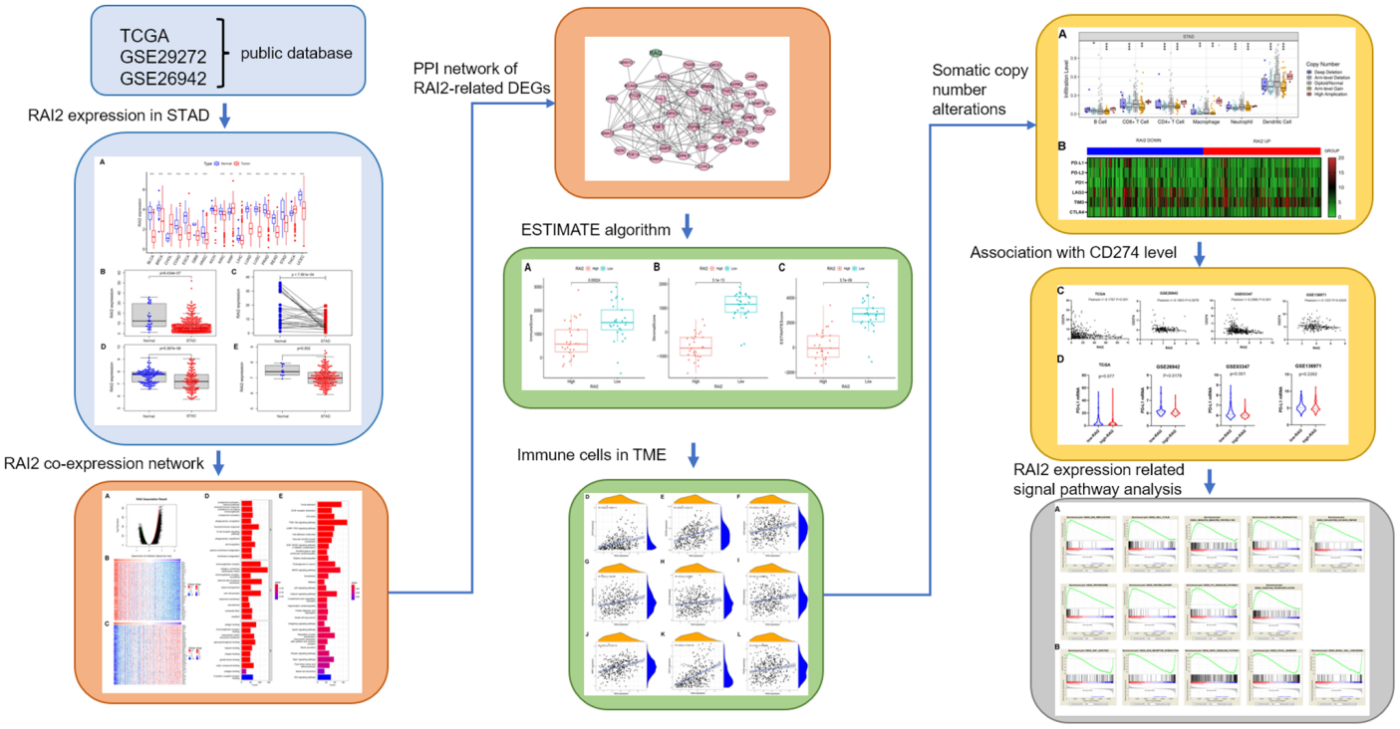
The workflow of this study.

Retinoic acid-induced 2 (RAI2) is an innovative tumor suppressor [7, 8] and as a newly discovered gene, little has been discovered regarding the way it works biologically. RAI2, located in the Xp22 region of the chromosome, expresses a 2.5 kb transcript and plays a pivotal role in the regulations controlling embryonic tissue development and cell growth [9]. However, there have been a few studies conducted on RAI2 in malignant tumorigenesis, progression, and metastasis. In 2015, Werner et al. [8] revealed for the first time that lower expression of RAI2 was an independent prognostic factor for breast cancer patients. Further investigations pointed out that RAI2 promoted the expression of various cellular differentiation genes by interacting with the transcription factor carboxyl-terminal binding protein 2 (CtBP2), which ultimately inhibited the early hematogenous metastasis of human breast cancer cells to bone marrow. Yan et al. [7] found that RAI2 inhibited the proliferation of colorectal cancer cells via blocking the AKT signalling pathway. The expression of RAI2 in human colorectal cancer is regulated by methylation in its promoter region, and RAI2 methylation is an indicator of poor prognosis for patients with colorectal cancer. More recently, Yang et al. [10] investigated the mechanisms of RAI2 expression and found that in bladder cancer the circRNA RBPMS-miR-330-3p signaling axis could regulate the expression of RAI2. Nonetheless, the activities of RAI2 in GC progression is mainly unknown. In the current study, the effect of RAI2 on GC growth and invasion was discovered and its potential mechanisms identified using various public databases analysis (Figure 1) and a sequence of *in vitro* experiments. We found that RAI2 could decrease the proliferation and invasion in GC cells through reducing PD-L1 expression and related to tumor-infiltrating lymphocytes (TILs), suggesting that it may be an innovative treatment option for GC patients.

## MATERIALS AND METHODS

### The expression analysis of RAI2 in GC

The expression of the RAI2 gene in GC was examined from public databases such as The Cancer Genome Atlas (TCGA) [11], Gene Expression Omnibus (GEO) [12], and GEPIA [13] databases. Differentially expressed genes (DEGs) related to the transcription of RAI2 in stomach adenocarcinoma (STAD) were analyzed on the basis of the LinkedOmics [14] (http://www.linkedomics.org/login.php) functional modules.

### GO and KEGG pathway analysis

Gene Oncology (GO) and Kyoto Encyclopedia of Genes and Genomes (KEGG) pathway enrichment analyses were performed for obtaining the featured genes by PCA algorithm with “clusterProfiler”, “ggplot2” “enrichplot”, “org.Hs.eg.db”, using R package [15]. Terms were regarded as significantly enriched only with both *P* < 0.05 and *q* < 0.05.

### Immunohistochemistry staining

Immunohistochemical (IHC) staining assay was conducted in paraffin-embedded tissues from GC patients to examine RAI2 protein levels. There were 20 cases of GC and paired samples of normal stomach tissues. The IHC staining was performed as previously reported [16]. In brief, the sectioned tissue slides were deparaffinized, and blocked with normal bovine serum. After that, the slides were stained with anti-RAI2 primary antibody (Cell Signaling Technology, 97857S, batch no. 09/2019) at 4°C overnight, then incubated with goat anti-rabbit IgG at room temperature for 1.5 h and with DAB solution for 15 min, and counterstained with hematoxylin solution using REAL EnVision kit (DAKO, USA, K5007). Each tissue slide was assessed on the basis of the staining intensity and the percentage of cells. The H-score was performed as reported [16]. The H-score value scopes between 0 and 300. A *t*-test was used to compare the RAI2 expression in GC tissues and the paracancer tissues. All tissues from GC patients who had not received radiotherapy or chemotherapy before surgery were collected between May 2017 to June 2018 in the Second Affiliated Hospital of Soochow University and the consent forms were signed by all patients.

### ESTIMATE algorithm and identification of stromal and immune groups

Estimation of Stromal and Immune cells in MAlignant Tumor tissues using Expression data (ESTIMATE algorithm) was performed to get the stromal and immune scores on the basis of the expression of two independent sets to consist of 141 genes which represented the degree of tumor stromal and immune infiltrations [17]. The analysis methods were consolidated in R package in R 3.6.0 as we previously reported [18].

### PPI network construction of differentially expressed genes

STRING website (https://cn.string-db.org/) was employed to build the PPI network of DEGs [15]. Cytoscape version 3.6.1 was then used for reconstruction, and nodes were selected to build a PPI network with a confidence level greater than 0.95.

### Tumor-infiltrating immune cells (TICs) profile analysis

TIC profile was estimated by the “e1071” and “preprocessCore” R packages in R 3.6.0 and CIBERSORT computational method for GC samples from the TCGA datasets was selected for subsequent analyses by filtering samples with *p* < 0.05. The content of each immune cell was calculated by assessing the expression of characteristic genes for an individual immune cell in each sample. The correlations between RAI2 mRNAs and immune cells infiltration of patients with GC were evaluated using Tumor Immune Estimation Resource (TIMER) database (https://cistrome.shinyapps.io/Timer) [19].

### Gene set enrichment analysis

The mechanisms of RAI2 expression on the progression of GC was assessed using GSEA software (v4.0.3) [20, 21]. HALLMARK gene set was gained from the MSigDB database V7.2. The Nominal *p*-value (NOM *p* < 0.05) was regarded as obviously enriched.

### Cell culture and siRNA transfection

Human GC cell line MGC803 was obtained from the Cell Bank of the Chinese Academy of Sciences. The cells were cultured in Dulbecco’s modified Eagle medium (DMEM, Hyclone, SH30022) containing 10% fetal bovine serum (FBS, Gibco, 10099141C) at 37°C in an atmosphere with 5% CO_2_. The MGC803 cells were transfected with RAI2 siRNA or siRNA control using Lipoctamine3000 (Invitrogen, L3000015) based on the manufacturer’s instructions. After 48 h, the transfected cells were harvested for the following experiments.

### Alamarblue assay

Cell proliferation was assessed by the Alamarblue assay as previously described [22, 23]. In brief, cells were added into 96-well plates. After 22 h, 46 h, 70 h, or 94 h incubation period, the cells in every well were mixed with 10 μL of Alamarblue solution (Invitrogen, DAL1025). The plates were incubated for another 2 h and assessed by a SpectraMax M5 multi-detection reader (Molecular Devices Corporation, CA, USA).

### Cell migration and invasion assays

Transwell migration assay was performed by Transwell Chambers (Corning, 3422). The invasion assay was performed using chambers coated with Matrigel matrix (1:8 dilution, BD Bioscience, 354234) [23, 24]. Approximately 1.0 × 10^5^ cells were added into the upper chambers with DMEM, and 0.5 ml DMEM with 10% FBS was subjected to the lower chambers. After 24 h, the migrated or invaded cells of the lower chamber were stained using Wright-Giemsa staining kit (Nanjing Jiancheng, D010), and assessed.

### Statistical method

Data are presented as mean ± S.D by using SPSS 18.0. The test of significance was done with the Student’s *t*-test. *P* values of less than 0.05 were considered to have significant statistical significance.

## RESULTS

### Reduced expression of RAI2 in GC

**Figure 2:**
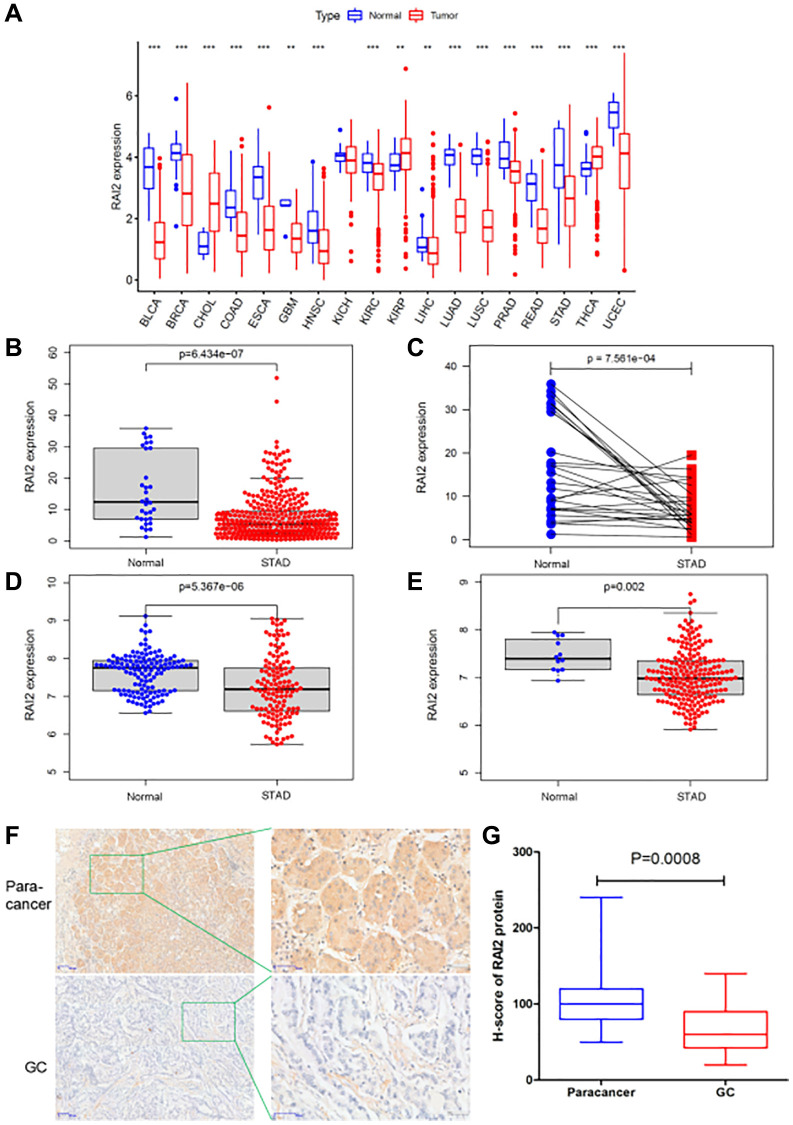
**The expression level of RAI2 in GC tissues.** (**A**) The expression of RAI2 in various kinds of tumors from TCGA database. (**B**) The expression of RAI2 between normal tissues and GC tissues from TCGA database. (**C**) RAI2 expression in paired samples of GC from TCGA database. (**D**, **E**) The expression of RAI2 mRNA in normal tissues and GC tissues from GS29272 (**D**) and GSE26942 (**E**) in GEO datasets, respectively. (**F**) The immunohistochemistry (IHC) assay was conducted to examine the expression of RAI2 protein in 20 GC tissues and paracancer tissues (50×, 200×). (**G**) The statistical analysis of RAI2 expression in GC tissues and paracancer tissues. ^**^P < 0.01.

To identify the level of RAI2 expression in distinct types of tumours, we initially analyzed the TCGA database. The research revealed that RAI2 was down-regulated in most tumors, including bladder urothelial carcinoma (BLCA), stomach adenocarcinoma (STAD), breast invasive carcinoma (BRCA), colon adenocarcinoma (COAD), glioblastoma multiforme (GBM), esophageal carcinoma (ESCA), head and neck squamous cell carcinoma (HNSC), prostate adenocarcinoma (PRAD), among others (Figure 2A). Through reference searching, we found that RAI2 was seldom examined in GC. Thus, we evaluated the levels of RAI2 in TCGA STAD datasets. RAI2 mRNA expression in GC decreased greatly, according to the results (Figure 2B, *P* < 0.01). Furthermore, we validated the low expression of RAI2 in tumor samples by analyzing 27 paired samples from TCGA STAD datasets (Figure 2C, *P* < 0.01). In addition, by analyzing the GEO datasets GSE29272 and GSE26942, we also confirmed the down-regulation of RAI2 in GC (Figure 2D, 2E, *P* < 0.01). The expression of RAI2 in GC tissues and paracancer tissues were further verified by IHC staining. As illustrated in Figure 2F, the expression of RAI2 protein was mainly localized in the cytoplasm, which was yellow to brown granular. And the expression of RAI2 protein in GC was obviously lower than that in normal adjacent tissues (Figure 2G). The outcomes pointed out RAI2 levels were significantly lower in GC tissues than in normal tissues.

### RAI2 co-expression network suggested potential functions in GC

**Figure 3:**
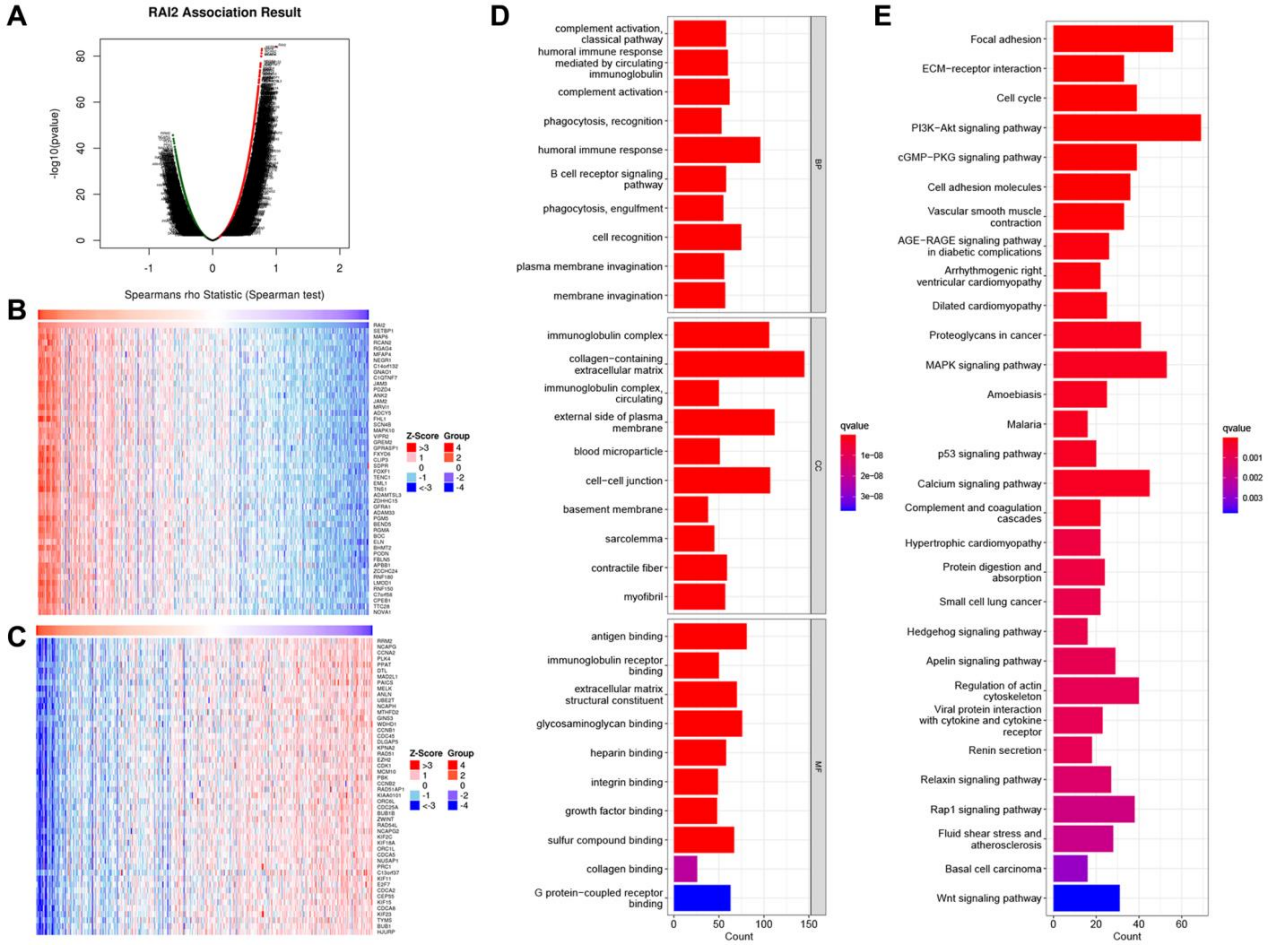
**Differential expression genes related to RAI2 in STAD from TCGA.** (**A**) Identification of significantly differential expressed genes associated to RAI2 through Pearson test. (**B**, **C**) Top 50 genes associated with RAI2 in STAD. Red: positive correlation genes; Green: negative correlation genes. (**D**, **E**) Bar plot for GO enrichment (**D**) and KEGG pathway (**E**) of RAI2 associated genes in STAD.

To find out the biological functions of RAI2 in GC, we analyzed the RAI2 co-expression network in the STAD cohort applying LinkedOmics. The analysis showed that 636 genes (dark red dots) correlated greatly with RAI2, while 468 genes (dark green dots) exhibited a significant negative correlation with RAI2 (false discovery rate, FDR < 0.01) (Figure 3A). The heat map displays the top 50 genes closely associated with RAI2 expression and the top 50 genes not associated with RAI2 expression (Figure 3B, 3C). RAI2 expression was significantly in association with SET binding protein 1 (SETBP1, positive rank #1), which was involved in DNA replication. On the other hand, ribonucleotide reductase regulatory subunit M2 (RRM2), which catalyzes the formation of deoxyribonucleotides, was shown to be the most negatively correlated gene to RAI2. RRM2 was synthesized in a cell-cycle-dependent manner. GO term annotation analysis confirmed that RAI2-related genes primarily engaged in humoral immune response, collagen-containing extracellular matrix, and antigen-binding (Figure 3D). Also, KEGG pathway analysis implied that the RAI2 co-expressed genes were clustered in pathways such as focal adhesion, ECM-receptor interaction, PI3K-Akt signaling pathway, MAPK signaling pathway, cell cycle pathway and among others (Figure 3E). Furthermore, the PPI network of the significant RAI2-related genes was constructed using the STRING database. The resulting network of RAI2-related genes was built and visualized using Cytoscape software, and it contained 40 nodes in the mRNA network which formed 161 connections (Supplementary Figure 1). This data proved that RAI2 played a key part in cell adhesion/invasion, immune regulation, DNA replication, and cell cycle in GC.

### Correlation analysis of RAI2 expression and immune infiltration level

**Figure 4:**
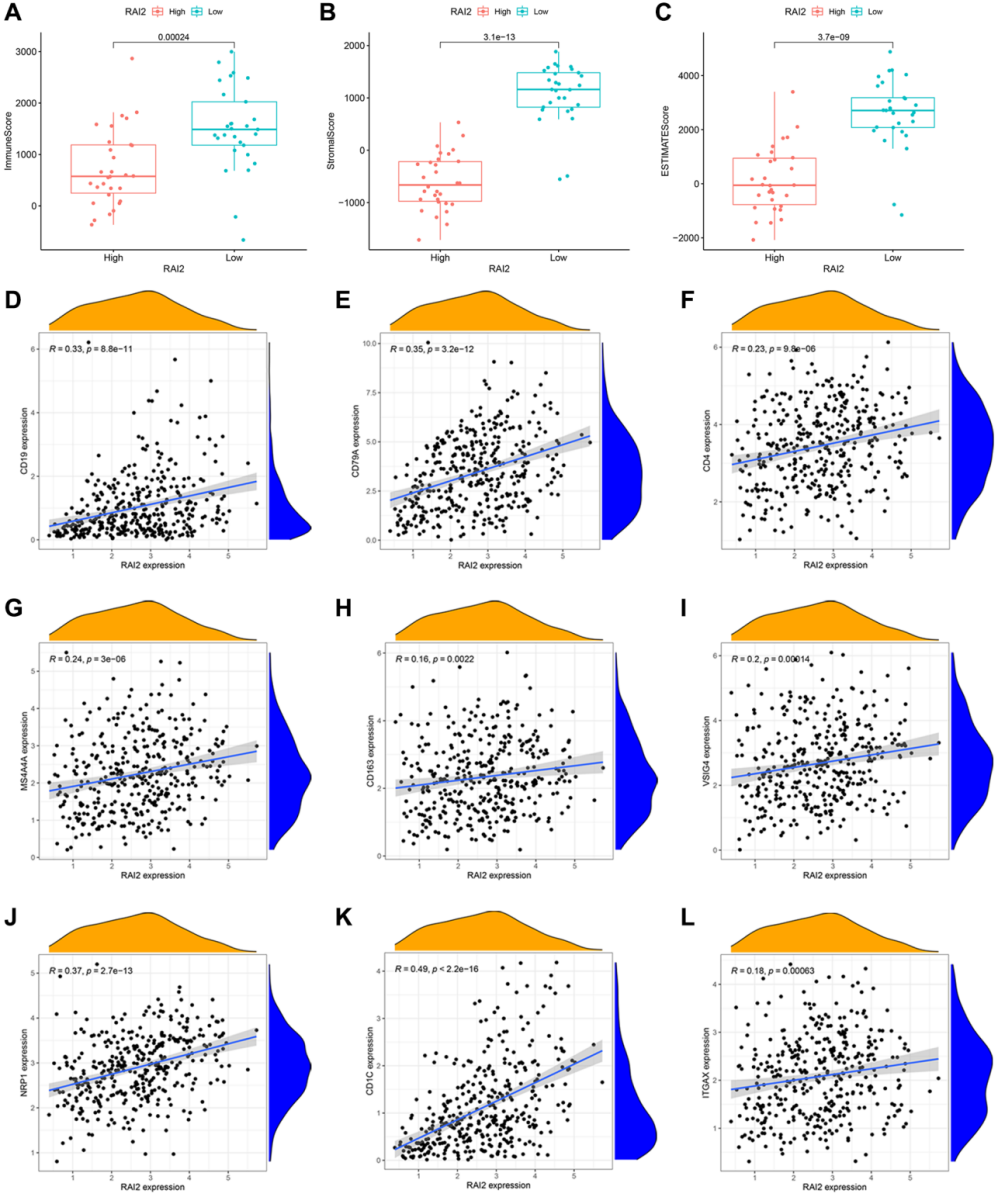
**Correlation between RAI2 expression and tumor immune microenvironment in STAD samples.** (**A**–**C**) Associations between the expression levels of RAI2 and immune score (**A**), stromal score (**B**) and ESTIMATE score (**C**). (**D**–**L**) Correlation between surface markers of immune cells and RAI2 expression. (**D**, **E**) B cell, (**F**) CD4+ T cell, (**G**–**I**) M2 macrophage, (**J**–**L**) dendritic cell.

To explore the penetration of immune cells and stromal cells within tumour sites, we computed the immune score and stromal score of gastric samples in the TCGA STAD dataset by way of the ESTIMATE algorithm (Figures 4A, 4B). Moreover, the ESTIMATE score, which represents tumor purity, was obtained by adding the immune and stromal scores (Figure 4C). These results indicated that samples with lower RAI2 expression contained more immune and stroma cells (Figure 4A–4C). Subsequently, we inquired into the specific types of immune cells in the tumor microenvironment based on the cell markers. The expression of B cells, CD4+ T cells, M2 macrophages, and dendritic cells were correlated significantly with RAI2 expression level (Figure 4D–4L).

### Correlation of RAI2 level and immune checkpoint

**Figure 5:**
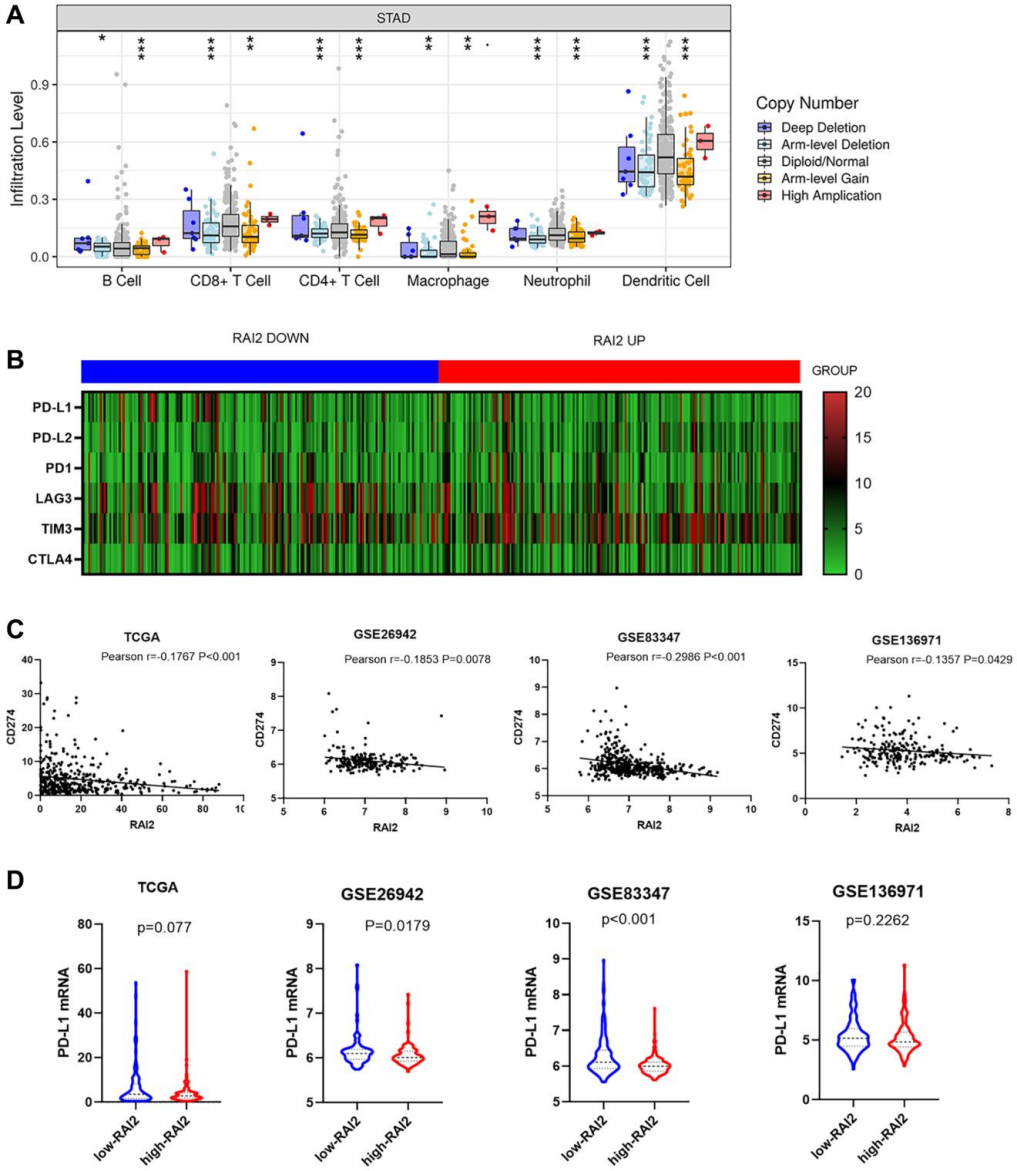
**RAI2 expression was significantly associated with PD-L1 expression.** (**A**) The effect of RAI2 copy number variation on the infiltration level of B cells, CD4+ T cells, macrophages and dendritic cells in STAD. (**B**) The heatmap of immune checkpoints based on RAI2 expression. (**C**) The correlation between RAI2 expression and CD274 expression in different databases. (**D**) CD274 mRNA expression decreased by RAI2 up-regulation.

In this research, the TIMER website was used to examine the causal connection between tumour infiltration levels and specific somatic copy number alterations for RAI2. The results revealed the RAI2 copy number variation (CNV) was highly associated with B cells, CD4+ T cells, CD8+ T cells, neutrophils, macrophages, and dendritic cells (Figure 5A). With the recent interest in immune-checkpoint inhibitor therapy research, we wanted to investigate the relationship between RAI2 and immune checkpoint in STAD. Through the TIMER website, a negative correlation was found between CD274 and RAI2, however, there was no statistical significance found among PD-L2, PD1, LAG3, TIM3, and CTLA4 (Figure 5B). Furthermore, the association between the up-regulation of RAI2 expression level and decreased CD274 level was observed by analyzing GC samples from the TCGA, GSE26942, GSE83347, and GSE136971 datasets (Figure 5C). Additionally, up-regulation of RAI2 mRNA level also associated with decreased CD274 mRNA level throughout the four databases (Figure 5D). These findings provided preliminary evidence that RAI2 may be a novel predictor in immunotherapy in GC.

### RAI2 expression-related signal pathway analysis

**Figure 6:**
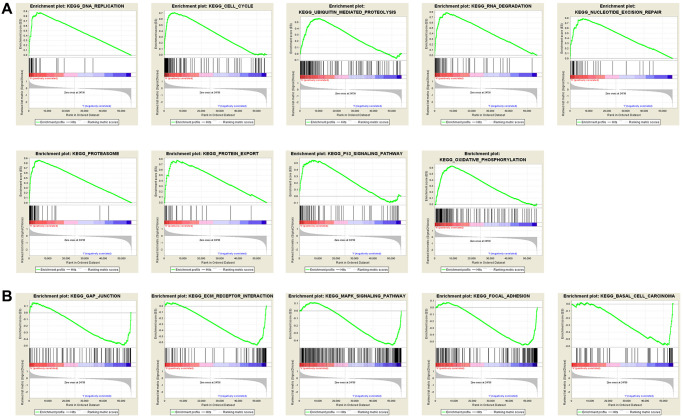
**Annotation of KEGG pathway among the top 30 samples with the highest and lowest RAI2 expression via multiple GSEA analysis.** (**A**) Pathways positively correlated with RAI2 expression. (**B**) Pathways negatively correlated with RAI2 expression.

To investigate the potential signal pathways RAI2 is involved in, we compared the RAI2 high expression set to the low expression set through GSEA to recognize the signal pathways activated in GC. These findings indicated that the majority of RAI2-overexpressed genes had enhanced expression in cell phenotype-related pathways, such as cell cycle, ECM receptor interaction, protein export, and classic tumor-associated pathway including the P53 signaling pathway (Figure 6A). In contrast, the RAI2 low-expression group showed enrichment in pathways including the MAPK signaling pathway, GAP junction, focal adhesion, and ECM receptor interaction pathway (Figure 6B). These pathways mainly activated tumor aggression and metastasis and the regulation of the tumor-related signal pathways caused a change in cell proliferation. These results, therefore, suggest that RAI2 might impact the growth, migration, and invasion of GC cells.

### RAI2 knockdown promoted the growth and invasion of GC cells

**Figure 7:**
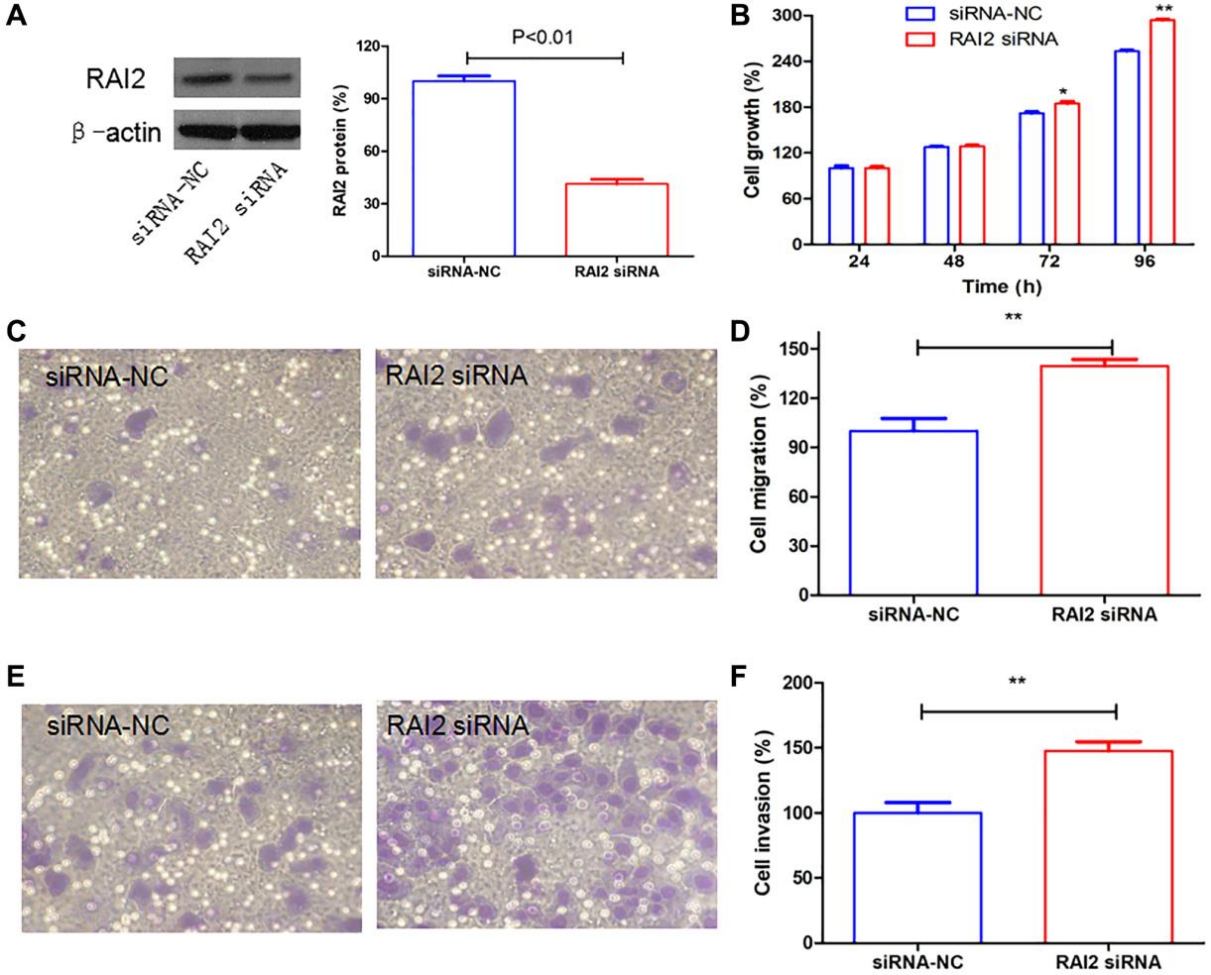
**RAI2 knockdown promoted the growth, cell migration, and invasion in GC cells.** (**A**) The knockdown efficiency of RAI2 in GC cell line MGC803 using siRNA. (**B**) The AlamarBlue assay of cell proliferation in MGC803 cells transfected with siRNA NC or RAI2 siRNA. (**C**–**F**) The Transwell cell migration and invasion abilities were assessed in MGC803 cells using RAI2 siRNA. ^**^P < 0.01, ^*^P < 0.05.

In order to comprehend and explain the significance of RAI2 in GC progression, we used specific siRNA to RAI2 to knock down its expression (Figure 7A). We performed the Alamarblue assay, which established that the knockdown of RAI2 promoted the cell proliferation of GC cells MGC803 (Figure 7B). Furthermore, migration and invasion tests on Transwell suggested that RAI2 knockdown could considerably increase the migratory and invasive abilities of tumor cells (Figure 7C–7F). These findings implied that RAI2 was an opposite controller of GC cell proliferation and invasion.

## DISCUSSION

Tumor metastasis is a complex process that includes a series of sequential processes such as the detachment of tumor cells from the primary tumor foci, crossing the interstitium, penetrating the stroma to invade blood vessels or lymphatic vessels, reaching distant organs or tissues with blood flow, colonizing and malignantly proliferating at distant sites to form clones, and growing into new blood vessels, and among others [25–30]. The molecular mechanisms involved in these processes are very complex. RAI2, a recently recognized tumour suppressor, is believed to have major parts in the progression and metastasis of quite a few of malignancies including breast cancer [8, 31], colorectal cancer [7, 32], bladder cancer [10], and prostate cancer [33]. However, the specific functions of RAI2 in the proliferation and invasion of GC cells have not been comprehensively investigated.

In the current research, we relied on bio-informatics to examine the expression of RAI2 in pan-cancer and discovered that RAI2 was abnormally expressed in the majority of malignancies, including GC. To further establish the relationship between RAI2 and GC, RNA-seq data in TCGA and GEO were analyzed, respectively. We established that the RAI2 levels were significantly decreased in GC, and this was further confirmed by means of an IHC staining assay on 20 pairs of GC and paracancer tissue samples.

To acquire a comprehensive comprehension of the contribution of RAI2 in the advancement of GC, we assessed the genes whose expression was strongly linked with RAI2 expression in GC. Much like to RAI2, the expression of these genes is aberrant in GC. These genes and RAI2 may constitute a regulatory network to promote the occurrence, development, and metastasis of GC. Through GSEA analysis, we analyzed the enrichment of the co-expressed genes with RAI2. The results showed that the regulatory network mainly focused on pathways such as focal adhesion, GAP junction, focal adhesion and ECM receptor interaction pathway, immune regulation, PI3K-Akt signaling pathway, MAPK signaling pathway, cell cycle pathway, and DNA replication. Consistent with the pathological characteristics of extremely proliferative malignancies such as GC, these findings supported the hypothesis. To further examine the functional position of RAI2 in the development and metastasis of GC, we performed multiple *in vitro* experiments in the GC cells, MGC803, and silenced RAI2 expression through siRNA transfection. The results indicated that knockdown of RAI2 promoted the proliferation, migration, and invasion of GC cells, confirming that RAI2 is essential for the *in vitro* maintenance of the tumorigenic activity of GC cells.

Tumor immunotherapy has become a promising therapeutic approach that controls and eradicates tumours by resetting and sustaining the tumor-immune cycle and reinstating the body's usual anti-tumor immune reaction [34]. This therapeutic approach includes strategies such as monoclonal antibody-based immune checkpoint inhibitors, cancer vaccines, therapeutic antibodies, and cell therapy. In the past few years, the emergence of tumor immunotherapy has proved its effectiveness of anti-tumor activity in treating a wide range of cancers, like melanoma, non-small cell lung cancer, GC, and other solid tumors. Recently, many studies have highlighted a correlation between cancer progression, metastasis, and tumor immune infiltration [34–36]. However, the relationship between RAI2 and immune regulation in GC remains poorly understood. Therefore, we investigated the effect of RAI2 in GC in the context of immunity. Our analysis through public data mining revealed that GC samples with lower RAI2 expression contained more immune and stroma cells. Moreover, the expression of B cells, CD4+ T cells, M2 macrophages, and dendritic cells were significantly associated with RAI2 expression levels. The results presented argue that RAI2 could play an attribute in GC immune regulation. Programmed death ligand 1 (PD-L1, also known as CD274) expression has been proposed as one of the pan-cancer biomarkers for immunotherapy including GC, breast cancer, lung cancer, etc. [37, 38]. Furthermore, several studies have reported that PD-L1 is implicated in other signaling roles, such as pro-survival, glycolytic metabolism, and reducing mTOR activity [39]. Thus, we tested the connections between RAI2 and PD-L1 expressions. Analysis of the TCGA STAD and GEO datasets revealed a negative relation between the expression of RAI2 and PD-L1 in GC samples. Moreover, up-regulation of RAI2 mRNA levels connected also with decreased PD-L1 mRNA levels across the analyzed datasets. These findings suggest that low expression of RAI2 in GC had a relationship with elevated TIL density and PD-L1 expression levels. This effort raises intriguing questions for further research about the predictive power of RAI2 in patients with GC when they undergo therapy with immune checkpoint blockade treatments.

**Figure 8:**
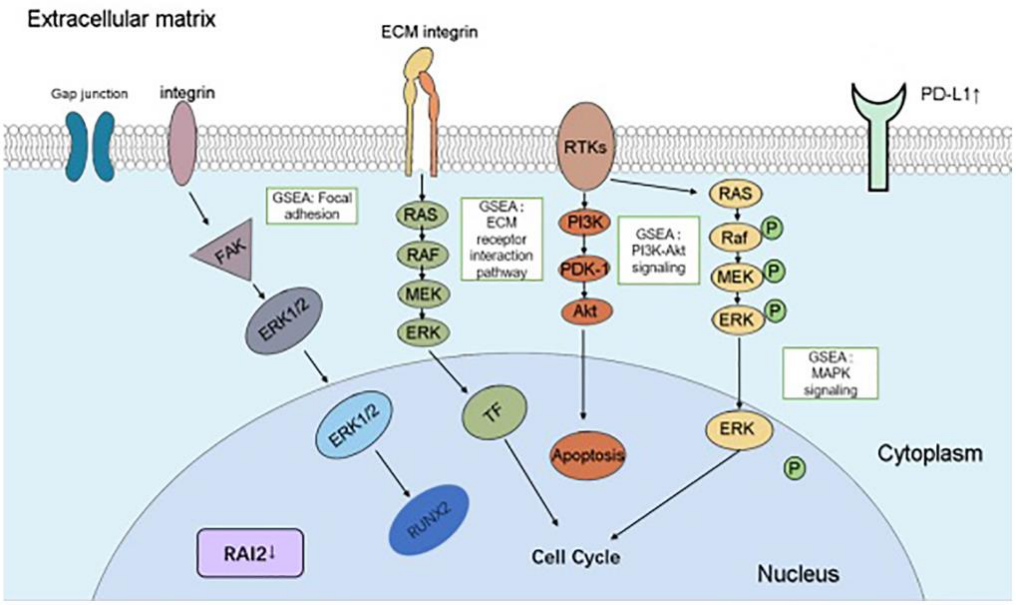
**A schematic diagram depicting the roles and mechanisms of RAI2 in GC cells.** The boxes indicated the abnormal pathways affected by RAI2 in GC cells, including “focal adhesion”, “ECM receptor interaction pathway”, “PI3K-Akt signaling” and “MAPK signaling”. Aberrant expressed genes by RAI2 were indicated in the figure.

In summary, our research delivered evidence at multiple levels for the crucial role of RAI2 in GC growth and invasion and its capacity as a predictor in immunotherapy for GC through reducing PD-L1 expression in conjunction with being relevant to TILs (Figure 8). These results noticed an avenue for the research and development of anti-cancer methods in GC.

## Supplementary Materials

Supplementary Figure 1
