# Supplementary material for: RAI2 acts as a tumor suppressor with functional significance in gastric cancer
Source: Aging (Albany NY). 2023 Oct 25;15(21):11831–44. doi: 10.18632/aging.205135 (PMC10683588; doi:10.18632/aging.205135)
Supplement: Supplementary Figure 1 [file aging-15-205135-s001.pdf]

SUPPLEMENTARY FIGURE

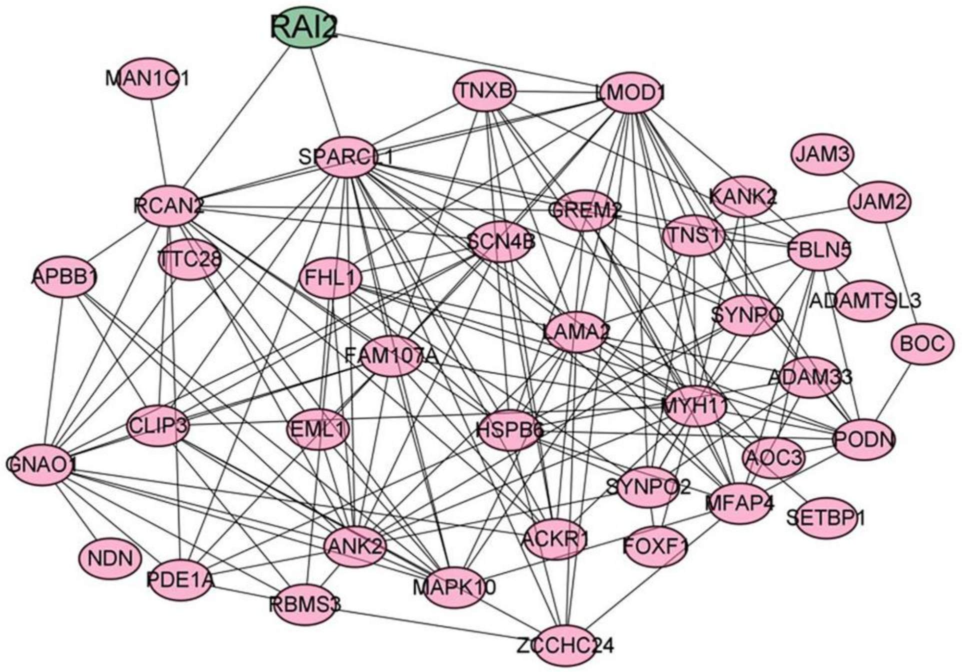

Supplementary Figure 1. Protein-protein interaction networks of differential expression genes related to RAI2 in GC.
